# Inflammatory bowel disease as a disorder of an imbalance between pro- and anti-inflammatory molecules and deficiency of resolution bioactive lipids

**DOI:** 10.1186/s12944-015-0165-4

**Published:** 2016-01-13

**Authors:** Undurti N. Das

**Affiliations:** UND Life Sciences, 2020 S 360th St, # K-202, Federal Way, WA 98003 USA; BioScience Research Centre and Department of Medicine, GVP Hospital, Gayatri Vidya Parishad College of Engineering Campus, Madhurawada Visakhapatnam, 530 048 India

**Keywords:** Inflammatory bowel disease, Lipoxins, Polyunsaturated fatty acids, Cytokines, Free radicals, Resolvins, Protectins

## Abstract

The inflammatory process seen in inflammatory bowel disease (IBD) is due to excess production of pro-inflammatory cytokines interleukin-1 (IL-1), IL-6, tumor necrosis factor-α (TNF-α), interferons (IFNs), macrophage migration inhibitory factor (MIF), HMGB1 (high mobility group B1) and possibly, a reduction in anti-inflammatory cytokines IL-10, IL-4, and transforming growth factor-β (TGF-β). These pro-inflammatory molecules lead to increased production of reactive oxygen species (ROS) including nitric oxide resulting in target tissue damage. I propose that inadequate production of inflammation resolving molecules lipoxins, resolvins, protectins, maresins and nitrolipids that suppress inflammation, ROS production, enhance wound healing and have cytoprotective properties results in inappropriate inflammation, delay in healing/repair process and so target tissue/organ damage continues in IBD. Hence, suggested therapeutic approach could include administration of stable synthetic analogues of lipoxins, resolvins, protectins, maresins and nitrolipids. This implies that measuring urine, stool and plasma levels of lipoxins, resolvins, protectins, maresins and nitrolipids may be used to detect the onset, progression and response to treatment of IBD.

## Background

Although genetic, environmental and sex hormonal factors have been implicated in the pathogenesis of autoimmune diseases, including inflammatory bowel disease (IBD), it is known that pro-inflammatory cytokines, nitric oxide (NO), free radicals, a deranged immune system, a deficient anti-oxidant defenses, Toll-like receptors and microbiota play a significant role both in the initiation and perpetuation of the inflammatory process observed. Once the inflammatory process is triggered, this leads to the production of a variety of pro-inflammatory molecules and possibly, a reduction in the elaboration of anti-inflammatory molecules. This imbalance between the pro- and anti-inflammatory molecules would ultimately cause target tissue/organ damage seen in these conditions. Despite many advances in the understanding of the pathobiology of IBD, it is still not clear what triggers the onset of IBD and which factors are needed to induce resolution of the same.

## Hypothesis

**Fig. 1:**
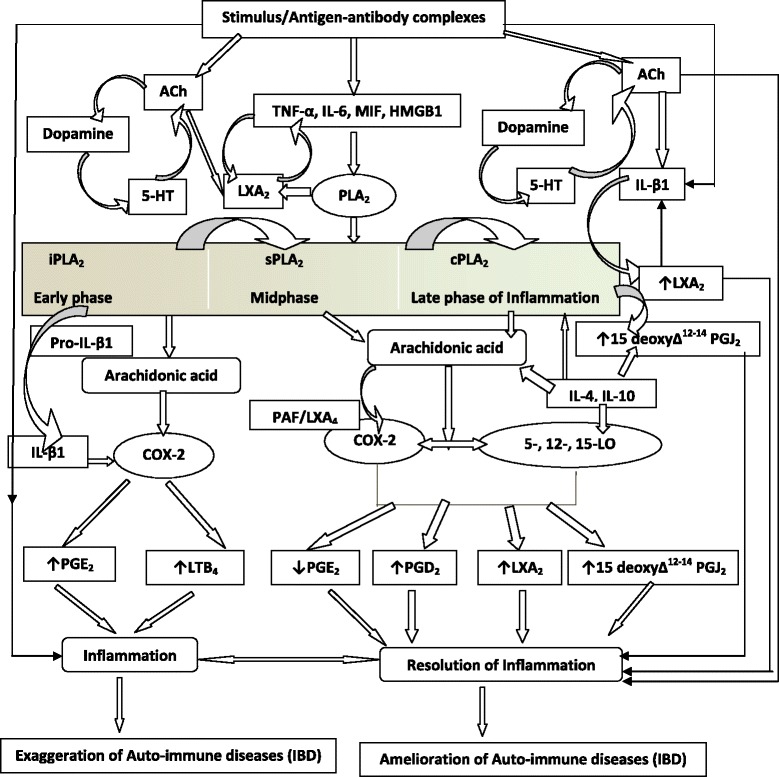
Scheme showing role of pro and anti-inflammatory bioactive lipids in IBD. For easy understanding only LXA_4_ has been depicted in the figure. It is to be noted that resolvins, protectins, maresins and nitrolipids may follow the same pathway as that of LXA_4_ from the respective precursors (EPA and DHA: eicosapentaneoic acid and docosahexaenoic acid respectively). Possible interaction among cholinergic anti-inflammatory pathway (Ach), serotonin, dopamine and LXA_4_ is also depicted in the figure. There are three classes of phospholipases that control the release of AA and other PUFAs: calcium-independent PLA_2_ (iPLA_2_), secretory PLA_2_ (sPLA_2_), and cytosolic PLA_2_ (cPLA_2_). Each class of PLA_2_ is further divided into isoenzymes for which there are 10 for mammalian sPLA_2_, at least 3 for cPLA_2_, and 2 for iPLA_2_. During the early phase of inflammation, COX-derived PGs and lipoxygenase-derived LTs initiate exudate formation and inflammatory cell influx. TNF-α causes an immediate influx of neutrophils concomitant with PGE_2_ and LTB_4_ production, whereas during the phase of resolution of inflammation an increase in LXA_4_ (lipoxin A_4_), PGD_2_ and its product 15deoxyΔ^12-14^PGJ_2_ formation occurs that induces resolution of inflammation with a simultaneous decrease in PGE_2_ synthesis that stops neutrophil influx and enhances phagocytosis of debris. Thus, there appears to be two waves of release of AA and other PUFAs: one at the onset of inflammation that causes the synthesis and release of PGE_2_ and a second at resolution for the synthesis of anti-inflammatory PGD_2_, 15deoxyΔ^12-14^PGJ_2_, and lipoxins that are necessary for the suppression of inflammation. Thus, COX-2 enzyme has both harmful and useful actions by virtue of its ability to give rise to pro-inflammatory and anti-inflammatory PGs and LXs. Increased type VI iPLA_2_ protein expression was found to be the principal isoform expressed from the onset of inflammation up to 24 h, whereas type IIa and V sPLA_2_ was expressed from the beginning of 48 h till 72 h while type IV cPLA_2_ was not detectable during the early phase of acute inflammation but increased progressively during resolution peaking at 72 h. This increase in type IV cPLA_2_ was mirrored by a parallel increase in COX-2 expression. The increase in cPLA_2_ and COX-2 occurred in parallel, suggesting a close enzymatic coupling between these two. Thus, there is a clear-cut role for different types of PLA_2_ in distinct and different phases of inflammation. Selective inhibition of cPLA_2_ resulted in the reduction of pro-inflammatory molecules PGE_2_, LTB_4_, IL-1β, and platelet-activating factor (PAF). Furthermore, inhibition of types IIa and V sPLA_2_ not only decreased PAF and LXA_4_ (lipoxin A_4_) but also resulted in a reduction in cPLA_2_ and COX-2 activities. These results suggest that sPLA_2_-derived PAF and LXA_4_ induce COX-2 and type IV cPLA_2_. IL-1β induced cPLA_2_ expression. This suggests that one of the functions of IL-1 is not only to induce inflammation but also to induce cPLA_2_ expression to initiate resolution of inflammation. Synthetic glucocorticoid dexamethasone inhibited both cPLA_2_ and sPLA_2_ expression, whereas type IV iPLA_2_ expression is refractory to its suppressive actions. Activated iPLA_2_ contributes to the conversion of inactive proIL-1β to active IL-1β, which in turn induces cPLA_2_ expression that is necessary for resolution of inflammation. LXs, especially LXA_4_ inhibit TNF-α-induced production of ILs; promote TNF-α mRNA decay, TNF-α secretion, and leukocyte trafficking and thus attenuated inflammation. Though the proposal presented has focused mainly on LXA_4_, it may be mentioned here that there could be a significant role for other anti-inflammatory bioactive lipids such as resolvins, protectins, maresins and nitrolipids in IBD. Thus, in all the places wherever LXA_4_ is mentioned, it may be assumed that resolvins, protectins, maresins and nitrolipids also have an important role to play along with LXA_4_

I propose that continued inflammatory events seen in IBD are due to failure of the resolution of inflammation. Thus, the balance between inflammation and resolution is disturbed more in favor of pro-inflammatory events and/or failure of resolution inducing molecules to be produced at the most appropriate time leading to non-resolution of inflammation. In other words, even after the inciting agent responsible for the initiation of inflammation is removed; inappropriate inflammation continues simply because resolution failed to occur. This leads to delay in the healing/repair process and so tissue/organ damage continues. This may explain as to why even when these patients are continuing to take anti-inflammatory and immunosuppressive medicines, target organ damage continues. In view of this, it is suggested that institution of pro-resolution-inducing agents could induce remission and restore normal physiological function of the target tissues/organs in IBD and other autoimmune diseases. Such endogenous pro-resolution-inducing molecules include: lipoxins, resolvins, protectins, maresins, nitric oxide, nitrolipids, 15 deoxyΔ^12–14^ PGJ_2_, PGD_2_, anti-inflammatory cytokines such as IL-4, IL-10, and some polyunsaturated fatty acids (PUFAs) (see Fig. 1).

Based on this hypothesis, it is suggested that progression and flares of IBD are not only due to increased production of pro-inflammatory molecules IL-6, TNF-α, MIF (macrophage migration inhibitory factor), HMGB1 (high mobility group box 1), free radicals and lipid mediators such as prostaglandins (PGs), leukotrienes (LTs) but also as a result of decreased formation and release of anti-inflammatory molecules: IL-4, IL-10, TGF-β, and lipoxins, resolvins, protectins, maresins and nitrolipids.

### Lipoxin A_4_ is detectable in urine

LXA_4_, generated by lipoxygenase (LO) transformation of AA possess potent anti-inflammatory activity in vivo, and temporal biosynthesis of LXA_4_, concurrent with spontaneous resolution, has been observed during exudate formation [1–3]. LXs, resolvins, protectins and maresins, which are anti-inflammatory bioactive lipids formed from polyunsaturated fatty acids: arachidonic acid (AA), eicosapentaenoic acid (EPA) and docosahexaenoic acid (DHA), are detectable in the plasma. Recently, it was shown that urine contains LXA_4_ and exercise significantly increased urinary excretion in healthy volunteers [4, 5], suggesting that alterations in the urinary excretion of LXA_4_ can used as a reflection of changes in LXA_4_ formation to monitor changes in the inflammatory process.

Urinary levels of LXA_4_ was decreased while that of cysLTs (cysteinyl leukotrienes) increased in volunteers aged from 26 to over 100 years leading to an imbalance of the LXA_4_/cysLTs ratio that is an index of the endogenous anti-inflammatory potential [6, 7]. These results suggest that endogenous anti-inflammatory mechanisms become less efficient with age that could result in increased susceptibility to inflammatory disorders with advancing age. Similar to their measurement in urine, lipoxins, resolvins and protectins can also be measured in stools and plasma.

These results imply that measurement of plasma, urinary and stool LXs and LTs could be used as a marker of inflammatory process in IBD. In this context, it is noteworthy that anti-inflammatory cytokines IL-4 and IL-10 trigger the conversion of AA, EPA and DHA to lipoxins, resolvins, protectins and maresins suggesting a mechanism by which they are able to suppress inflammation [8].

### LXA_4_ and LTs ratio determines the degree of inflammation

In animal models of glomerulonephritis and other renal pathologic states, LTs exerted adverse effects in the glomerulus. LTB_4_ augments neutrophil infiltration, and LTC_4_ and LTD_4_ mediate potent vasoconstrictor effects on the glomerular microcirculation. Selective blockade of the 5-lipoxygenase pathway produced a significant amelioration of the deterioration of renal hemodynamic and structural parameters. On the other hand, 15-S-hydroxyeicosatetraenoic acid (15-S-HETE), the immediate product of arachidonate 15-lipoxygenase, and the lipoxins, which are produced by sequential 15- and 5- or 5- and 12-lipoxygenation of AA are also generated in the course of glomerular injury that antagonize leukotriene-induced neutrophil chemotaxis and lipoxin A_4_ antagonized the effects of LTD_4_ and LTC_4_ on the glomerular microcirculation. Thus, the contrasting effects of 5- and 15-lipoxygenase products represent endogenous pro- and anti-inflammatory influences that ultimately determine and regulate the extent and severity of glomerular inflammation [9–12].

These results are in favor of the proposal that deficiency of anti-inflammatory bioactive lipids may play a significant role in autoimmune diseases including IBD.

### PUFAs and lipoxins bind to GPCR and suppress inflammation

Monocytes and macrophages express an extensive repertoire of G protein-coupled receptors (GPCRs) that regulate inflammation and immunity [13]. PUFAs, especially AA, EPA and DHA and their metabolites such as eicosanoids, lipoxins, resolvins, protectins and maresins function as agonists at a number of G protein-coupled receptors (GPCRs) that play a significant role in leukocyte recruitment and inflammation. G protein-coupled receptor 120 (GPCR120) that functions as a ω-3 fatty acid receptor/sensor that when stimulated with ω-3 fatty acids (EPA and DHA) induced broad anti-inflammatory effects in monocytic RAW 264.7 cells and in primary intraperitoneal macrophages that were abrogated by GPR120 knockdown [14]. Thus, PUFAs and their anti-inflammatory bioactive lipids may suppress inflammation in autoimmune diseases by acting on GPCRs.

Based on the preceding discussion, I suggest that increased production of lipoxins, resolvins, protectins and maresins at sites of inflammation in IBD may suppress the disease process. Failure to produce adequate amounts of these anti-inflammatory lipoxins, resolvins, protectins, maresins and nitrolipids could render the inflammatory process to continue unabated. Hence, methods designed to enhance the production of lipoxins, resolvins, protectins and maresins and/or administration of synthetic analogues of lipoxins, resolvins, protectins and maresins will aid in the prevention and management of IBD and other autoimmune diseases. This proposal is supported by the recent report [15] that β-Cell destruction produced by multiple low-doses streptozotocin was completely prevented in fat-1 mice (transgenic fat-1 mice carry a *C. Elegans* gene, fat-1, encoding an n-3 fatty acid desaturase catalyzing the conversion of n-6 to n-3 PUFAs and hence, these animals have high tissue levels of n-3 PUFAs) that was associated with increased levels of the anti-inflammatory lipoxin A_4_ and the 18-hydroxyeicosapentaenoic acid, precursor of the anti-inflammatory resolvin E_1_.

### Testing the hypothesis

The hypothesis presented here can be tested by performing relevant in vitro, in vivo and human studies. (1) In vitro studies could be performed using colon epithelial cells. For instance, one can use HCoEpiC cells from ScienCell Research laboratories (USA) which are isolated from human colon tissue. These cells can process and present antigens to T cells in vitro, and can be stimulated to express HLA class II and intercellular adhesion molecules in vivo [16]. They also respond to a broad array of cytokines with altered gene expression and growth characteristics [17]. These HCoEpiC cells could be exposed to LPS (lipopolysaccharide) and various pro- and anti-inflammatory cytokines and their ability to secrete pro- and anti-inflammatory bioactive lipids such as prostaglandins, leukotrienes, thromboxanes, lipoxins, resolvins, protectins, maresins and nitrolipids could be measured. It is anticipated that in response to pro-inflammatory stimuli, HCoEpiC cells would produce increased amounts of prostaglandins, leukotrienes, thromboxanes and IL-6 and TNF-α and reduced concentrations of anti-inflammatory bioactive lipids such as lipoxins, resolvins, protectins and maresins and IL-10, IL-12 and TGF-β. In contrast, when HCoEpiC cells are supplemented with anti-inflammatory cytokines would secrete higher amounts of lipoxins, resolvins, protectins, maresins and nitrolipids and less of prostaglandins, leukotrienes and thromboxanes.

(2) In vivo studies could include colonic instillation of the hapten, trinitrobenzene sulphonic acid (TNB) that induces ulceration and inflammation which can persist for 3 weeks [18]. Another IBD model that could be studied includes the guinea pig acetic acid-induced colonic inflammation model that shows characteristics of IBD including PMN infiltration, edema, ulceration and necrosis [19]. Both acute and chronic colitis can also be induced by feeding female Swiss-Webster mice with 5 % DSS (dextran sulfate sodium 30,000–40,000 mol wt) for 5 or 7 d, respectively [20]. This IBD model could be used to study prophylaxis, prophylaxis plus therapy and therapeutic effect of lipoxins, resolvins, protectins, nitrolipids (all as singly or in combination) during the acute and chronic phase of the disease and therapeutically during the chronic phase of the disease. Thus, a variety of IBD models could be used to study the balance between pro- and anti-inflammatory bioactive lipids and changes in their concentrations during the onset of the disease, progression of the disease and remission of IBD to understand the dynamics of their changes in various phases of the disease. In these animal models of IBD, plasma, tissue (colonic secretions and colonic tissue) and urinary levels of prostaglandins, leukotrienes, thromboxanes, lipoxins, resolvins, protectins, maresins, nitrolipids and various cytokines could be measured to ascertain their role in the disease process. Similarly, lipoxins, resolvins, protectins, maresins and nitrolipids can be administered (including their more stable synthetic analogues) to know their protective action against IBD.

(3) Most important studies that need to be performed to firmly establish the role of bioactive lipids will be to measure the plasma, stool and urinary levels (and if possible colonic concentrations) of anti-inflammatory lipoxins, resolvins, protectins, maresins and nitrolipids and pro-inflammatory prostaglandins, leukotrienes and thromboxanes and various cytokines in subjects who are having acute, chronic and subacute features of IBD. If the hypothesis presented here is correct, it is expected that plasma, colonic, urinary and stool concentrations of lipoxins, resolvins, protectins, maresins and nitrolipids and anti-inflammatory cytokines will be low whereas those of pro-inflammatory bioactive lipids and cytokines will be high during the acute and subacute and chronic phases of IBD (though the degree of elevation could be variable). It is likely that all pro- and anti-inflammatory bioactive lipids and cytokines need not be altered and the alteration may be seen only in one or more than one of the biomolecules. If such a study is in support of the hypothesis presented here, then it is worthwhile to study the preventive and curative action of more stable and synthetic analogues of lipoxins, resolvins, protectins, maresins and nitrolipids in patients with IBD.

### Implications of the hypothesis

If the hypothesis presented here is true, it will usher in a new era in our approach to IBD, which predisposes to the development of colon cancer, in the form of use of lipoxins, resolvins, protectins, maresins and nitrolipids in its prevention and treatment. It is possible that more stable and orally active synthetic analogues of lipoxins, resolvins, protectins, maresins and nitrolipids could be developed for IBD. Furthermore, if such an approach of use of lipoxins, resolvins, protectins, maresins and nitrolipids in the prevention and management of IBD becomes successful, it will pave way to treat other autoimmune diseases such as lupus, rheumatoid arthritis, multiple sclerosis and type 1 diabetes mellitus.

## Conclusions

Based on the preceding discussion, it is likely that administration of stable synthetic analogues of lipoxins, resolvins, protectins and maresins may form a new approach in the prevention and treatment of IBD. Measurement of urine, stool and plasma levels of lipoxins, resolvins, protectins, maresins and leukotrienes would help in determining whether IBD is in remission, continuing or resolving. For instance, if the levels of lipoxins, resolvins, protectins, maresins and leukotrienes revert to normal it implies that IBD is in entering remission phase or is in remission; and if the levels of lipoxins, resolvins, protectins, maresins and nitrolipids are lower than normal and those of leukotrienes are high it indicates that the disease is continuing and/or not responding to the therapy instituted. In those subjects who are on therapy for IBD if the levels of lipoxins, resolvins, protectins, maresins and nitrolipids are not increasing and/or if the levels of leukotrienes are increasing it indicates that the disease is continuing and remission is yet to set in. On the other hand, if the plasma, urine and stool levels of lipoxins, resolvins, protectins, maresins and nitrolipids are increasing and those of leukotrienes are decreasing it indicates that the underlying IBD is entering remission phase and that the patient is responding to the therapy instituted. Thus, serial measurements of lipoxins, resolvins, protectins, maresins, nitrolipids and leukotrienes will give an indication as to the progress and response to therapy. It is likely that changes in the plasma, urine and stool levels of lipoxins, resolvins, protectins, maresins, nitrolipids and leukotrienes will precede the clinical remission or progression IBD. It is also envisaged that by determining the plasma, stool and urinary levels of lipoxins, resolvins, protectins, maresins and nitrolipids in those with IBD, one will be able to determine which of these bioactive lipids is needed for appropriate therapy. For instance, in those with IBD who show only a decrease in lipoxins need to be given lipoxins; those with deficiency of resolvins need only resolvins; those who show low levels of protectins need to get only protectins and similarly those who have decreased levels of maresins need to be administered only maresins. It is anticipated though lipoxins, resolvins, protectins, maresins and nitrolipids are all anti-inflammatory and resolution inducing molecules, all patients with IBD may not have deficiency of all these five bioactive lipids. It is likely that patients with IBD may show deficiency of only one, two, three or all five of these molecules. This heterogeneity in the deficiency of these bioactive lipid molecules could due to genetic heterogeneity in the synthesis and action of pro-resolution bioactive lipids (namely lipoxins, resolvins, protectins and maresins), polymorphism of 5-, 12- 15-lipoxygenases and cyclooxygenases. Such genetic heterogeneity and polymorphism of lipoxygenases and cyclooxygenases may explain innumerable variations in the histological and clinical presentation of IBD and their response to therapies employed.

The recent observation that the cholinergic anti-inflammatory pathway based on vagus nerve activity regulates macrophage and dendritic cell responses in the spleen through alpha-7 nicotinic acetylcholine receptor (a7nAChR) signaling and IBD patients have dysautonomia with decreased vagus nerve activity, dendritic cell and T cell over-activation [21] can also be attributed to alterations in the production of lipoxins, resolvins, protectins and maresins. For instance, it was reported that rats with metabolic syndrome have ineffective inflammation-resolving mechanisms that was associated with 120 % decrease in plasma LXA_4_ levels [22]. These results suggest a close interaction between the cholinergic anti-inflammatory pathway and lipoxins, resolvins, protectins and maresins. Furthermore, it was reported that electroacupuncture at the sciatic nerve controlled systemic inflammation by inducing vagal activation leading to the production of dopamine in the adrenal medulla. Dopamine inhibited cytokine production via dopamine type 1 (D1) receptors and D1 receptor agonists suppressed systemic inflammation and rescued mice from polymicrobial peritonitis. These results suggest that sciatic and vagus nerves modulate the production of catecholamines [23]. Since acetylcholine is able to enhance the production of lipoxins, resolvins, protectins and maresins (and possibly, nitrolipids), it is likely that dopamine is also able to enhance the production of these anti-inflammatory bioactive lipids. Though no studies have been performed with serotonin and its possible influence on the production of lipoxins, resolvins, protectins, maresins and nitrolipids, I propose that a close interaction exists between them. It is known that serotonin plays a significant role in the inflammatory events of IBD [24]. Studies showed that nicotine, which mediates its actions via α7-nicotine acetylcholine receptor (α7-nAChR), attenuated both IL-1β and 5-hydroxytryptamine (5-HT, serotonin)-evoked Ca^2+^ transients [25], suggesting that cholinergic anti-inflammatory pathway has a regulatory role on serotonin release and its actions. These evidences indicate that anti-inflammatory bioactive lipids, anti-inflammatory cholinergic pathway, serotonin and dopamine interact among them to regulate immune response and inflammation and thus, ultimately in the pathogenesis of IBD.

Furthermore, LXA_4_ seems to have a negative regulatory role on cholinergic regulatory pathway by elaborating nitric oxide. This implies that once the cholinergic anti-inflammatory pathway has reached its optimum, its action is negated by acetylcholine-induced LXA_4_ generation so that undesirable immunosuppression does not set in. In view of these interactions, administration of adequate amounts of lipoxins, resolvins, protectins and maresins are expected to be of benefit in the prevention and management of IBD.

## References

[1] Levy BD, Clish CB, Schmidt B, Gronert K, Serhan CN (2001). Lipid mediator class switching during acute inflammation signals in resolution. Nat Immunol.

[2] Serhan CN, Maddox JF, Petasis NA, Akritopoulou-Zanze I, Papayianni A, Brady HR (1995). Design of lipoxin A_4_ stable analogs that block transmigration and adhesion of human neutrophils. Biochemistry.

[3] Das UN (2010). Current and emerging strategies for the treatment and management of systemic lupus erythematosus based on molecular signatures of acute and chronic inflammation. J Inflammation Res.

[4] Romano M, Luciotti G, Gangemi S, Marinucci F, Prontera C, D’Urbano E (2002). Urinary excretion of lipoxin A(4) and related compounds: development of new extraction techniques for lipoxins. Lab Invest.

[5] Gangemi S, Luciotti G, D’Urbano E, Mallamace A, Santoro D, Bellinghieri G (2003). Physical exercise increases urinary excretion of lipoxin A4 and related compounds. J Appl Physiol.

[6] Gangemi S, Pescara L, D’Urbano E, Basile G, Nicita-Mauro V, Davì G (2005). Aging is characterized by a profound reduction in anti-inflammatory lipoxin A4 levels. Exp Gerontol.

[7] Wu SH, Liao PY, Yin PL, Zhang YM, Dong L (2009). Inverse temporal changes of lipoxin A4 and leukotrienes in children with Henoch-Schönlein purpura. Prostaglandins Leukot Essent Fatty Acids.

[8] Katoh T, Lakkis FG, Makita N, Badr KF (1994). Co-regulated expression of glomerular 12/15-lipoxygenase and interleukin-4 mRNAs in rat nephrotoxic nephritis. Kidney Int.

[9] Nassar GM, Badr KF (1995). Role of leukotrienes and lipoxygenases in glomerular injury. Miner Electrolyte Metab.

[10] Papayianni A, Serhan CN, Phillips ML, Rennke HG, Brady HR (1995). Transcellular biosynthesis of lipoxin A4 during adhesion of platelets and neutrophils in experimental immune complex glomerulonephritis. Kidney Int.

[11] O’Meara YM, Brady HR (1997). Lipoxins, leukocyte recruitment and the resolution phase of acute glomerulonephritis. Kidney Int Suppl.

[12] Wu SH, Liao PY, Yin PL, Zhang YM, Dong L (2009). Elevated expressions of 15-lipoxygenase and lipoxin A4 in children with acute poststreptococcal glomerulonephritis. Am J Pathol.

[13] Lattin JE, Schroder K, Su AI, Walker JR, Zhang J, Wiltshire T (2008). Expression analysis of G Protein-Coupled Receptors in mouse macrophages. Immunome Res.

[14] Oh DY, Talukdar S, Bae EJ, Imamura T, Morinaga H, Fan W (2010). GPR120 is an omega-3 fatty acid receptor mediating potent anti-inflammatory and insulin-sensitizing effects. Cell.

[15] Bellenger J, Bellenger S, Bataille A, Massey KA, Nicolaou A, Rialland M (2011). High pancreatic n-3 fatty acids prevent STZ-induced diabetes in fat-1 mice: inflammatory pathway inhibition. Diabetes.

[16] Mayer L, Eisenhardt D, Salomon P, Bauer W, Plous R, Piccinini L (1991). Expression of class II molecules on intestinal epithelial cells in humans. Differences between normal and inflammatory bowel disease. Gastroenterology.

[17] Eckmann L, Jung H-C, Schuerer-Maly C-C, Panja A, Morzycka-Wroblewska E, Kagnoff MF (1993). Differential cytokine expression by human intestinal epithelial cell lines: regulated expression of interleukin-8. Gastroenterology.

[18] Boughton-Smith NK, Wallace JL, Morris GP, Whittle BJ (1988). The effect of anti-inflammatory drugs on eicosanoid formation in a chronic model of inflammatory bowel disease in the rat. Br J Pharmacol.

[19] Fretland DJ, Levin S, Tsai BS, Djurić SW, Widomski DL, Zemaitis JM (1989). The effect of leukotriene-B4 receptor antagonist, SC-41930, on acetic acid-induced colonic inflammation. Agents Actions.

[20] Murthy SN, Cooper HS, Shim H, Shah RS, Ibrahim SA, Sedergran DJ (1993). Treatment of dextran sulfate sodium-induced murine colitis by intracolonic cyclosporin. Dig Dis Sci.

[21] Munyaka P, Rabbi MF, Pavlov VA, Tracey KJ, Khafipour E, Ghia JE (2014). Central muscarinic cholinergic activation alters interaction between splenic dendritic cell and CD4+CD25- T cells in experimental colitis. PLoS One.

[22] Su X, Feng X, Terrando N, Yan Y, Chawla A, Koch LG (2013). Dysfunction of inflammation-resolving pathways is associated with exaggerated postoperative cognitive decline in a rat model of the metabolic syndrome. Mol Med.

[23] Torres-Rosas R, Yehia G, Peña G, Mishra P, del Rocio Thompson-Bonilla M, Moreno-Eutimio MA (2014). Dopamine mediates vagal modulation of the immune system by electroacupuncture. Nat Med.

[24] Ghia JE, Li N, Wang H, Collins M, Deng Y, El-Sharkawy RT (2009). Serotonin has a key role in pathogenesis of experimental colitis. Gastroenterology.

[25] Westerlund A, Björklund U, Rönnbäck L, Hansson E (2013). Long-term nicotine treatment suppresses IL-1β release and attenuates substance P- and 5-HT-evoked Ca^**2+**^ responses in astrocytes. Neurosci Lett.

